# Enhanced Mechanical Properties Via Boron‐Induced Multi‐Mechanism Strengthening in (NbTaTi_1.5_V)_100‐x_B_x_ Refractory High Entropy Alloys

**DOI:** 10.1002/advs.76257

**Published:** 2026-06-29

**Authors:** Da Wu, Bo Li, Yuzhi Shi, Cong Li, Eryong Liu, Xiaohu Hou, Yimin Gao, Tao Wu, Pucun Bai, Chenyu Liang

**Affiliations:** ^1^ State Key Laboratory For Mechanical Behaviour of Materials School of Materials Science and Engineering Xi'an Jiaotong University Xi'an People's Republic of China; ^2^ School of Materials Science and Engineering Xi'an University of Science and Technology Xi'an People's Republic of China; ^3^ School of Materials Science and Engineering Inner Mongolia University of Technology Hohhot People's Republic of China; ^4^ Instrumental Analysis Center of Xi'an Jiaotong University Xi'an Jiaotong University Xi'an People's Republic of China

**Keywords:** (NbTaTi_1.5_V)_100‐x_B_x_ alloys, compressive fracture strength, crack deflection, grain boundary strengthening, plastic deformation

## Abstract

In this study, a trace amount of boron was added to the NbTaTi_1.5_V RHEA to enhance its strength. Specifically, (NbTaTi_1.5_V)_100‐x_B_x_ alloys with varying boron contents were fabricated by ball milling and spark plasma sintering (SPS). With the addition of 1 at.% boron, the compressive fracture strength of the alloy increased by 21.3% relative to the B0 alloy, rising from 2975 to 3610 MPa. A compressive fracture strain of 14.8% was maintained. The microhardness increased from 881 ± 67 HV to 1060 ± 60 HV, corresponding to an improvement of 20.4%. The microstructure analysis indicated that the (NbTaTi_1.5_V)_100‐x_B_x_ alloys were composed of three fine‐grained phases, namely BCC phase, TiO phase and TiB phase. The addition of trace amounts of boron led to significant grain refinement, resulting in a uniform and refined microstructure in the sintered alloys. The average grain size of B1 alloy was only 0.91 µm, representing a 67% reduction compared with that of the B0 alloy (2.82 µm). This provided a favorable microstructural foundation for the synergistic strengthening of the alloys. The boron element contributed 48.2% of the yield strength enhancement in B1 alloy by grain boundary strengthening through grain refinement, which was the dominant strengthening mechanism. TiB and TiO particles promoted dislocation proliferation, resulting in an additional strengthening contribution of approximately 28.7%. Furthermore, the TiB phase enhanced resistance to plastic deformation by impeding dislocation slip through crack deflection. This synergistic multi‐mechanism strengthening strategy offers an effective approach for improving the mechanical properties of (NbTaTi_1.5_V)_100‐x_B_x_ alloys.

## Graphical abstract

A trace amount of boron is introduced into NbTaTi_1.5_V RHEA, forming a finegrained BCC+TiO+TiB microstructure. The TiB phase generates numerous dislocations, strengthening the alloy. The alloy exhibits 3610 MPa compressive fracture strength and 1060 HV microhardness, while retaining 14.8% fracture strain. Grain‐boundary strengthening constitutes the main share (48.2%) of the yield strength increment, with an additional contribution from dislocation strengthening.

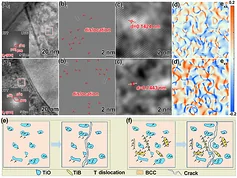

## 1 Introduction

High‐entropy alloys (HEAs) have attracted considerable attention due to their unique composition and microstructure [1, 2, 3]. HEAs typically consist of four or more principal elements in equiatomic or near‐equiatomic proportions. Therefore, HEAs are also known as multi‐principal element alloys [4, 5]. Compared with conventional alloys, HEAs offer greater compositional flexibility and design freedom [6, 7]. Since the discovery of HEAs, HEAs with FCC and BCC structures have been extensively investigated [8, 9, 10, 11]. In recent years, the rapid development of the aerospace industry has driven an increasing demand for structural materials with good mechanical properties. Refractory high‐entropy alloys (RHEAs) composed of refractory metals possess excellent mechanical properties and are attractive for aerospace application [12, 13]. However, RHEAs typically suffer from a pronounced strength‐ductility trade‐off, which limits their widespread industrial application.

Since its first report, the MoNbTaW RHEA has attracted considerable attention due to its excellent strength [14, 15]. However, the fracture strain of MoNbTaW RHEA at room temperature is only 2.6% [16]. To obtain a homogeneous and fine microstructure, Roh et al. [17] prepared NbMoTaW RHEA by mechanical alloying (MA) and spark plasma sintering (SPS). Tong et al. [18] synthesized an (NbTaW)_1‐x_Mo_x_ RHEA by arc melting, and improved the alloy properties via adjusting the Mo content. The above examples indicate that W/Mo‐containing RHEAs exhibit high compressive yield strength but suffer from limited ductility. To overcome the poor room‐temperature ductility of RHEAs, a series of alloys composed of intrinsically ductile elements (Hf, Nb, Ta, Ti, Zr, V) have been developed [19, 20, 21]. Among these, NbTaTi‐based RHEAs stand out as promising structural materials for extreme‐environment applications [22, 23]. The primary challenge for this alloy system is to further improve strength without compromising ductility. Vanadium plays a significant role in strengthening HEAs [24]. Consequently, the NbTaTiV RHEA stood out as an ideal candidate for a performance material. As‐cast NbTaTiV RHEA exhibits high compressive ductility (greater than 40%). However, its room‐temperature compressive yield strength of 965 MPa still offers considerable scope for improvement [25]. As‐cast RHEAs prepared by vacuum arc melting commonly have compositional segregation and dendritic structures [26, 27]. As is well known, *in‐situ* precipitated reinforcements usually have good dispersion within the matrix [28]. MA can effectively suppress microsegregation through forced solid‐state mixing. Subsequently, SPS yields a fine‐grained microstructure with a uniform composition. The mechanical properties of the alloy can be significantly enhanced through grain‐boundary strengthening [17].

To enhance the strength of NbTaTiV RHEAs, researchers have explored multiple strengthening strategies, including solid‐solution strengthening, grain refinement, and the introduction of secondary phases. In terms of composition and microstructure control, Guo et al. [29] fabricated an NbTaTiV RHEA with a homogeneous structure and fine grains by powder metallurgy (PM), attaining a compressive yield strength of 1.37 GPa while retaining a compressive ductility of 23%. Liu et al. [30] prepared a MoNbTaTiV RHEA by MA and SPS. The alloy exhibited an outstanding compressive yield strength of 2208 MPa together with a compressive plastic strain of 24.9% at room temperature. Liu et al. [31] adjusted the Ti content in Ti_x_VNbMoTa RHEA to achieve strengthening. The strengthening approaches described above primarily involve the addition of the intrinsically brittle element Mo, the adjustment of Ti content, and grain refinement. However, the essence of its strengthening mainly relies on the solid‐solution and grain‐boundary effects, and it still has not broken through the potential limitation of the strength‐plasticity trade‐off. The introduction of a second phase is also a powerful strategy for enhancing alloy strength [32, 33], and is commonly employed in other alloy systems [34, 35, 36]. Regarding RHEAs, Lu et al. [37] designed and fabricated an ultrafine‐grained Ti_40_Nb_15_Mo_30_(NbC)_15_ composite material by PM. Strengthening was achieved by introducing the ceramic reinforcing phase (Ti, Nb)C, which induces strong dislocation interactions and high resistance to dislocation motion. Zhao et al. [38] introduced trace boron to enhance the mechanical properties of Ti‐6Cr‐5Mo‐5V‐4Al. The boron addition precipitated TiB whiskers, resulting in a significant improvement in the alloy's mechanical performance. Maurício et al. [39] strengthened a TiNbZrTaMo RHEA by adding B_4_C powder to promote *in‐situ* formation of TiB and TiC reinforcements. However, this is still limited to the combination of carbides and borides. As a component of NbTaTiV RHEA, Ti is very sensitive to oxygen and is easy to form Ti oxides [40].

In our previous work [41], TiO particles were *in‐situ* generated in fine‐grained NbTaTiV alloy via MA+SPS. This confirmed the feasibility of single‐phase oxide strengthening in this alloy system. The above‐mentioned studies have all confirmed the effectiveness of a single type of ceramic phase or boride in strengthening RHEAs. However, they are still limited to a single type of reinforcement, and the potential of dual‐phase synergetic strengthening of RHEAs by oxides and borides has not been explored yet. Furthermore, by regulating the Ti content, NbTaTi_1.5_V RHEA achieves an excellent combination of high compressive yield strength and large compressive plastic strain [42]. Based on this finding, NbTaTi_1.5_V RHEA was selected as the research object. Considering the high affinity of Ti for O and B in NbTaTiV alloy, the alloy can be strengthened by *in‐situ* reactions that simultaneously generate TiO and TiB as secondary‐phase reinforcements. Different from previous studies that only employed a single oxide or a single boride, the dual‐phase design in this work aims to exert the dual strengthening effects of the two types of reinforcements. It breaks through the strength‐plasticity trade‐off dilemma often encountered in single‐phase strengthening strategies.

In this work, (NbTaTi_1.5_V)_100‐x_B_x_ alloys were fabricated by MA and SPS, and the effect of boron content on microstructure and mechanical performance was systematically investigated. Rapid densification via SPS effectively suppressed grain growth, yielding fine‐grained (NbTaTi_1.5_V)_100‐x_B_x_ alloys. The room‐temperature compressive properties of the alloys with different boron contents were studied, and a strengthening model was established to elucidate the strengthening mechanisms.

## 2 Experimental

### 2.1 Materials and Composites Preparation

**FIGURE 1:**
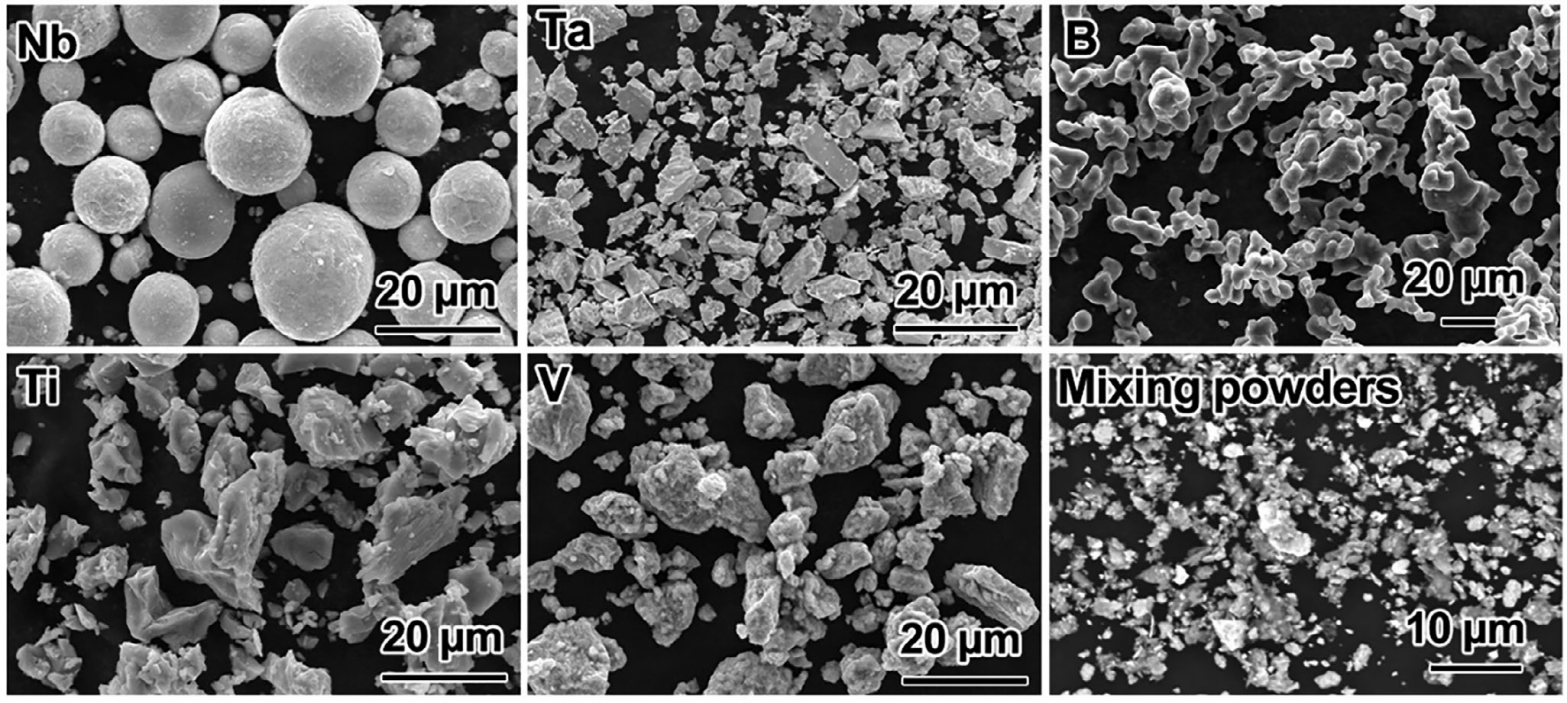
SEM images of the raw powders and mixed powders.

In this study, a series of (NbTaTi_1.5_V)_100‐x_B_x_ alloys with different boron contents were designed, where x represents the atomic percentage of boron, taking values of 0, 0.5, 1, 1.5, 2, and 2.5 at.%. For clarity, the alloys are designated according to their boron content. The corresponding alloy names are listed in Table 1. The preparation of these alloys started from raw powders of Nb, Ta, Ti, V, and B, with a particle size ranging from 10 to 50 µm. The morphologies of the raw powders and the mixed powders are shown in Figure 1. These powders were weighed according to the target compositions and then introduced into a planetary ball mill (model LC‐PBM‐6L, Shanghai Lichen Instrument Technology Co., Ltd.) for high‐energy ball milling. The ball milling process was conducted as follows. The weighed powders were transferred into a vacuum type ZrO_2_ milling vial with a rubber sealing ring. The milling media were ZrO_2_ balls with diameters of 3–6 mm, and the ball to powder mass ratio was set at 10:1. Subsequently, 30 mL of anhydrous ethanol was added as a process controlling agent. Then, the milling vial was evacuated for 3 min to remove the residual gas within. To limit temperature increase during milling, an intermittent milling procedure was adopted, involving 30 min of milling followed by a 10 min pause. The rotational speed was set at 300 rpm, and the total ball milling duration was 40 h. After milling, the powders were dried at 30°C for 2 h in a drying oven. The dried powders were subsequently sintered by SPS. Initially, a thin layer of graphite paper was affixed to the inner wall of the graphite mold to facilitate the demolding of the sintered sample. The powders were loaded into the graphite mold, and the filled mold was placed into the SPS furnace (SPS‐PM‐20/60, Shanghai Jinghua Technology Co., Ltd., China). Sintering was carried out under a vacuum of 2 × 10^−^^6^ Pa with an applied axial pressure of 50 MPa. To prevent temperature overshoot, a gradient heating method was employed: the sintering temperature was increased from room temperature to 800°C at a rate of 100°C/min and held for 2 min. Following this initial hold, the sample was heated to 1200°C at a rate of 100°C/min. Subsequently, the sample was heated to 1300°C at a rate of 50°C/min and maintained at 1300°C for 10 min. After sintering, the sample was cooled to room temperature in the furnace, yielding a cylindrical specimen with dimensions of Φ25.6 × 9 mm.

**TABLE 1:** Element content of (NbTaTi_1.5_V)_100‐x_B_x_ composites (at. %).

Name	Alloys	Nb	Ta	Ti	V	B
B0	NbTaTi_1.5_V	22.22	22.22	33.34	22.22	0
B0.5	(NbTaTi_1.5_V)_99.5_B_0.5_	22.1089	22.1089	33.1733	22.1089	0.5
B1.0	(NbTaTi_1.5_V)_99.0_B_1.0_	21.9978	21.9978	33.0066	21.9978	1.0
B1.5	(NbTaTi_1.5_V)_98.5_B_1.5_	21.8867	21.8867	32.8399	21.8867	1.5
B2.0	(NbTaTi_1.5_V)_98.0_B_2.0_	21.7756	21.8867	32.6732	21.8867	2.0
B2.5	(NbTaTi_1.5_V)_97.5_B_2.5_	21.6645	21.8867	32.5065	21.8867	2.5

### 2.2 Microstructural Characterization

The sintered alloys were processed into specific dimensions using wire electrical discharge machining, with the metallographic specimens measuring approximately 7 × 7 × 6 mm. The metallographic specimen was polished successively with sandpapers of #200, #400, #600, #800, #1000, #1500, and #3000. Subsequently, the specimen was polished to a mirror like finish using a SiO_2_ polishing solution and a silk polishing cloth. The density of each sintered alloy was measured using the Archimedes' displacement method. The microstructure and fracture morphology of the alloys were characterized by scanning electron microscopy (SEM, Quanta FEG 650, FEI, America). To analyze the phase constitution of the alloys with varying boron content, X‐ray diffraction (XRD) was performed using a diffractometer (XRD, Shaanxi Longrun International Trade Co., Ltd., China) with Cu‐Kα radiation (40 kV, 40 mA). The 2θ range was scanned from 20° to 90°. EBSD specimens underwent the same mechanical polishing procedure as the SEM specimens, followed by ion polishing at 12 kV for 50 min. Additionally, the phase distribution and grain size of the sintered alloys were examined using electron backscatter diffraction (EBSD) on a field‐emission scanning electron microscope (SEM JEOL 7100F). EBSD analysis was conducted using a scanning electron microscope operating at 20 kV with a step size of 0.06 µm. The raw data were post‐processed using AZtecCrystal software. The messy spikes were removed using the cleaning function of the software. Subsequently, particles with a grain area of less than 10 pixels were excluded to eliminate potential mis‐indexing artifacts. The high‐resolution microstructure of the sintered alloys was further investigated using transmission electron microscopy (TEM, Talos F200X). The preparation process of TEM specimen was as follows: First, a small disc with a diameter of 3.1 mm and a thickness of 0.5 mm was cut from the sintered specimen by wire‐cutting. Subsequently, the front and back sides of the specimen were polished with #240, #400, #800, and #1500 sandpapers in sequence to mechanically thin it down to approximately 80 µm. Finally, an EM RES102 ion‐thinning instrument was used for the final thinning to prepare a thin area suitable for TEM observation. The TEM data were analyzed using DigitalMicrograph software and the open‐source software Strain++.

### 2.3 Mechanical Property

Room‐temperature compression test was conducted on the sintered alloys at a constant crosshead speed of 0.5 mm/min using an electronic universal testing machine (AG‐XPLUS100KN, Shimadzu Corporation, Japan). The specimens were machined into cylindrical shapes with a diameter of 4 mm and a height of 6 mm by wire electrical discharge machining. To ensure the stability and reliability of the compression test data, each alloy was tested at least 3 times. Additionally, the microhardness of the sintered alloys with different boron contents was measured using a micro‐Vickers hardness tester (HV‐1000TPTA, LaiZhou Weiyi Experimental Machinery Manufacture Co., Ltd.). The test load of 200 g was applied for 15 s.

## 3 Results and Discussion

### 3.1 Phase Constitution and Microstructure of (NbTaTi_1.5_V)_100‐x_B_x_ Alloys

**FIGURE 2:**
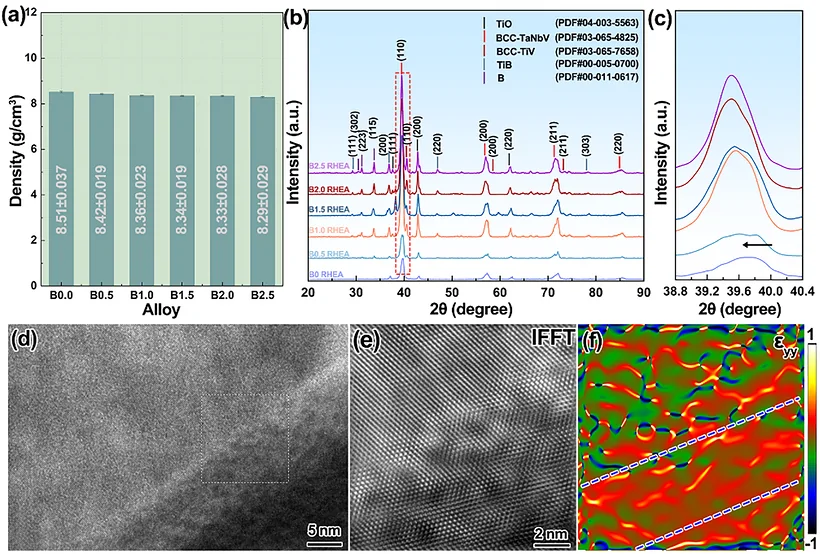
(a) Density of sintering alloy, (b) XRD patterns of (NbTaTi_1.5_V)_100‐x_B_x_ alloys, (c) enlarged XRD pattern of the selected area, (d) HRTEM of B_1_ alloy, (e) IFFT of (d), (f) ε_yy_ strain measured from (e).

Figure 2a shows the density changes of the sintered (NbTaTi_1.5_V)_100‐x_B_x_ alloys. As can be seen from the figure, with the increase in boron content, the alloy density gradually decreases. This is mainly due to the dilution effect of the light‐weight element boron on the overall density. However, the overall density difference is not significant. XRD patterns of (NbTaTi_1.5_V)_100‐x_B_x_ alloys with different boron contents are presented in Figure 2b. The B0 RHEA exhibits a typical BCC‐TaNbV crystal structure (PDF#03‐065‐4825) together with a small amount of TiO (PDF#04‐003‐5563). The diffraction peaks corresponding to the TiO phase are detected at 2θ = 37.1°, 42.9°, 62.3°. Be different from the B0 alloy, several new phases are identified in the other alloys with the addition of boron. These new phases include TiB (PDF#00‐005‐0700), BCC‐TiV (PDF#03‐065‐7658) and B (PDF#00‐011‐0617). The intensity of the TiB diffraction peaks in the B1 alloy does not increase with a further increase in boron content. This is likely because the availability of Ti is limited. The formation of the TiO phase has consumed a substantial amount of Ti, and a certain fraction of Ti remains dissolved in the BCC matrix. Nevertheless, as the boron content rises, the diffraction peaks of elemental boron in the boron‐containing alloys gradually strengthen. It is noteworthy that, in the B0 alloy, the (110) diffraction peak of the BCC‐TaNbV phase is observed at 2θ = 39.792°. Upon adding boron, this peak shifts slightly to the left, appearing at 39.552° in the boron‐containing alloys. This change is illustrated by the arrows in Figure 2c. This shift is likely attributable to tensile stress induced by the incorporation of boron, which causes the diffraction peak to move to lower 2θ values [41]. Figure 2d shows the high‐resolution transmission electron microscopy (HRTEM) image of B1 alloy. Figure 2e is IFFT image of the boxed area in Figure 2d, where significant lattice distortion can be observed. The strain distribution in this region is further analyzed using geometric phase analysis (GPA), and the result shows a non‐uniform strain field (Figure 2f), with tensile strain being dominant.

**FIGURE 3:**
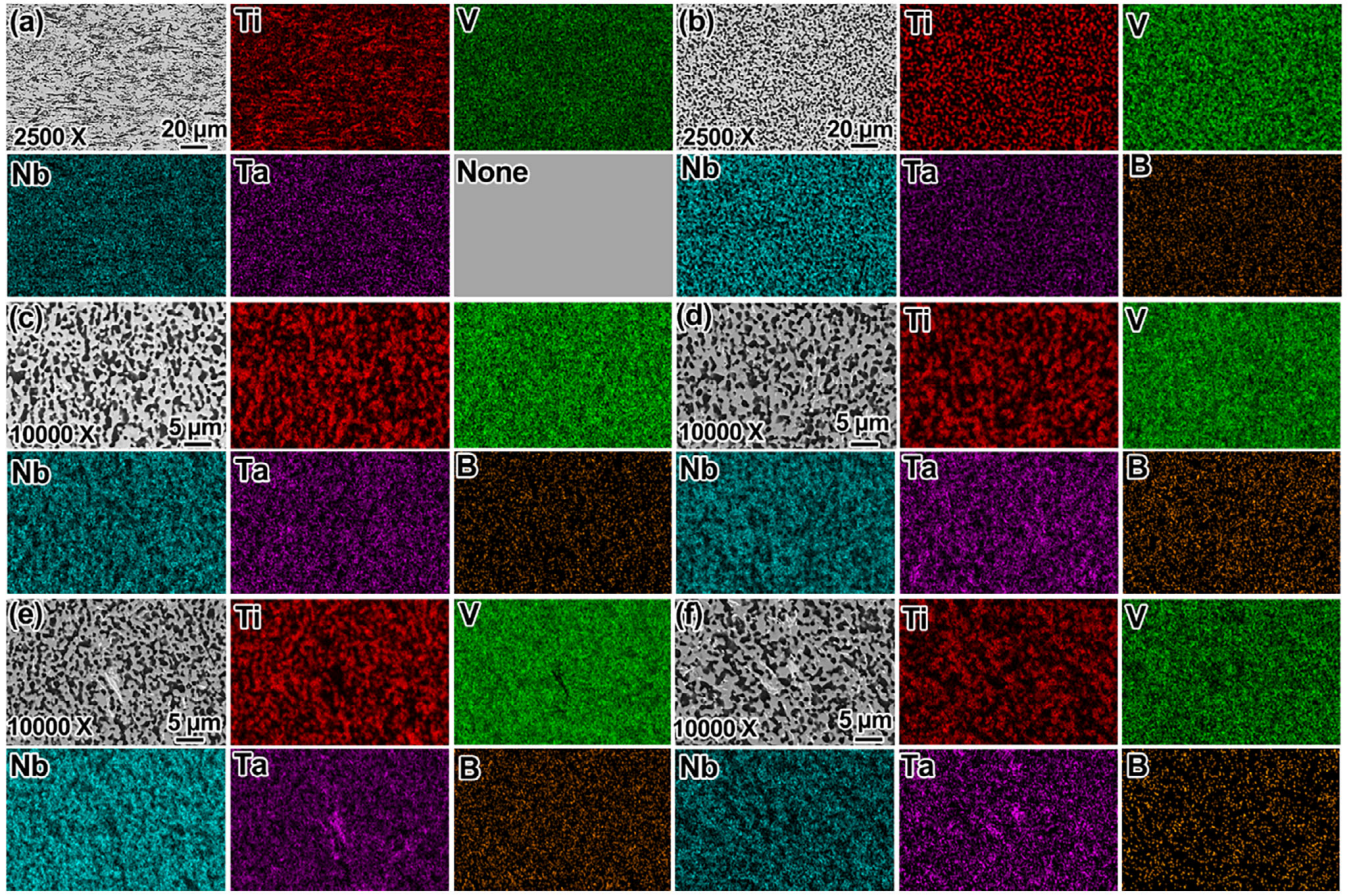
Microstructures of sintered (NbTaTi_1.5_V)_100‐x_B_x_ alloys with different boron content: (a) B0, (b) B0.5, (c) B1.0, (d) B1.5, (e) B2.0, (f) B2.5.

Analysis of the microstructure of the (NbTaTi_1.5_V)_100‐x_B_x_ alloys presented in Figure 3, it is concluded that the addition of boron is an effective strategy to refine the microstructure and enhance the overall performance of the alloys. Figure 3a shows the SEM microstructure of B0 alloy. It can be seen that the overall microstructure of the alloy is relatively uniform. According to the EDS surface scan, the dark regions in the microstructure correspond mainly to Ti‐enriched areas. The elements Nb, Ta, and V are evenly distributed in the gray matrix. With the addition of boron, the structure of the alloy is refined, as shown in Figure 3b. The EDS surface scan shows that the Ti element changes from a slender strip to a short strip. In the matrix, Nb, Ta, and V are uniformly distributed. Boron is obviously an ultralight element (*Z* = 5). Its extremely low K‐fluorescence yield (ω_K = 0.056% [43]) results in a very small X‐ray yield [44]. Moreover, the RHEAs matrix causes L/M line interferences from heavy elements and high background noise in the low‐energy spectrum. These factors further limit the EDS detection sensitivity of boron [45]. Furthermore, the low added boron content leads to a relatively weak contrast in the EDS surface scan signal. In the B1 alloy, the microstructure was clearly further refined, as shown in Figure 3c. However, the EDS elemental distribution maps indicate that the distribution characteristics of the corresponding elements did not change significantly. Only the EDS contrast for boron exhibits a slight increase, which can be attributed to the higher boron content. Figure 3d shows the microstructure of B1.5 alloy. It can be observed that as the boron content increases further, and the refinement effect tends toward saturation. The microstructure morphology is similar to that of B1 alloy. The microstructures of B2 and B2.5 alloys are shown in Figure 3e and Figure 3f, respectively. The microstructure no longer exhibits significant refinement, and the EDS signal of boron becomes increasingly pronounced as the content increases.

**FIGURE 4:**
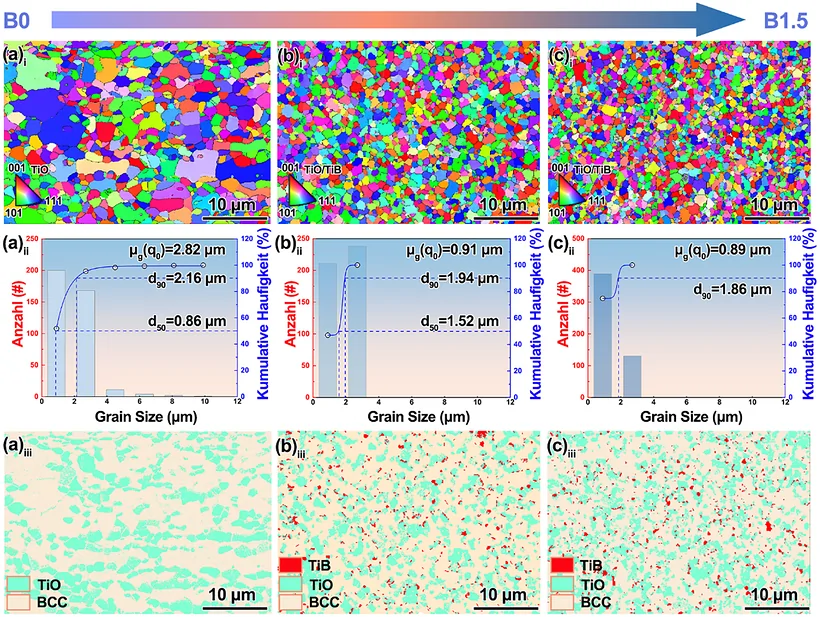
Electron back‐scattered diffraction (EBSD) of (NbTaTi_1.5_V)_100‐x_B_x_ alloys with different boron content: (a) B0, (b) B1, (c) B1.5. i, inverse pole figure (IPF). ii, grain size distribution. iii, phase distribution map.

The EBSD is used to further investigate the microstructural evolution of NbTaTi_1.5_V RHEAs with different boron content, and the results are shown in Figure 4. The IPF maps reveal a typical polycrystalline structure consisting of equiaxed grains with random orientations of various oriented grains (panel i of Figure 4a–c). With the addition of boron, the grains of the alloys have become significantly finer. To further quantify the grain‐refining effect of boron, the grain sizes of B0, B1.0, and B1.5 alloys were statistically analyzed. The results are presented in panel (ii) of Figure 4a–c. Statistical analysis shows that the average grain size of B0 alloy is approximately 2.82 µm (see panel ii of Figure 4a). In contrast, the average grain sizes of B1 and B1.5 alloys are 0.91 µm and 0.89 µm, respectively (panels ii of Figure 4b,c). The grain sizes of the B1 and B1.5 alloys are comparable. Relative to B0 alloy, B1 alloy exhibits a 67.7% reduction in average grain size, whereas B1.5 alloy shows a slightly greater reduction of 68.4%. This result indicates that boron addition leads to significant grain refinement in the NbTaTi_1.5_V RHEA. The phase distribution map (panel iii of Figure 4a) reveals that B0 alloy consists of BCC phase and TiO phase, with proportions of 76% and 24%, respectively. In the alloy with the addition of boron, the phase distribution map (panel iii of Figure 4b,c) shows that the BCC phase and TiO phase are the majority, with minor amounts of TiB phase also present. Further statistical analysis was performed on the volume fractions of each phase. In the B1 alloy the BCC matrix accounts for 68.8%. TiO oxide accounts for 26.2%. TiB boride accounts for approximately 5%. With the increase of boron content, the overall phase composition in B1.5 alloy remains nearly unchanged, exhibiting only a slight increase in TiB content to 5.2%. This suggests that boron primarily promotes the formation of TiB and exerts a limited influence on other phases.

**FIGURE 5:**
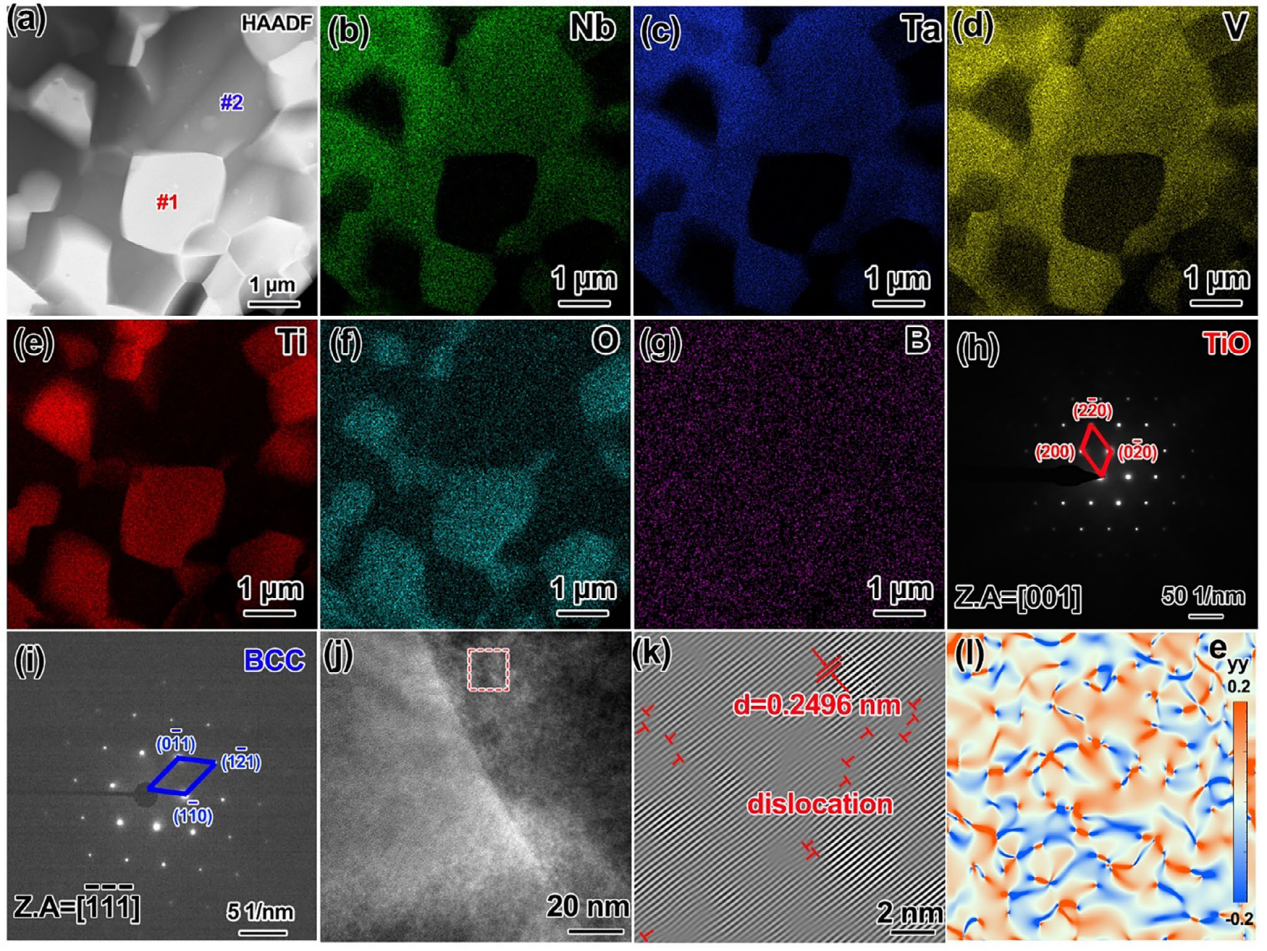
Microstructures of sintered B1 alloy: (a) HAADF TEM image. (b–g) EDS mappings of Nb, Ta, V, Ti, O, and B. (h) SAED at the position marked #1 in (a). (i) SAED at the position marked #2 in (a). (j) High‐resolution TEM (HRTEM) characterization, (k) inverse FFT (IFFT) HRTEM image of the selected rectangular part in (j). (l) Comparison of the atomic strain distribution in (k).

Figure 5a shows a high angle annular dark field (HAADF) image of the precipitated phase of sintered B1 alloy. The HAADF image reveals that the alloy consists mainly of equiaxed grains. Figure 5b–g show the EDS elemental maps for Nb, Ta, V, Ti, O, and B, respectively. As shown in Figure 5b–d, the dark‐gray grains in the HAADF image are enriched in Nb, Ta and V, whereas the bright regions (marked # in Figure 5a) contain mainly Ti and O. Trace amounts of boron are uniformly distributed (Figure 5g). Figure 5h shows the SAED pattern recorded from position #1 in Figure 5a. The indexing procedure confirms that this pattern corresponds to the TiO phase. Figure 5i displays the SAED pattern from position #2, and the indexing procedure identifies it as the BCC phase. Figure 5j shows a highresolution TEM image. Figure 5k presents the corresponding inverse fast fourier transform (IFFT) image of the TiO phase, in which a high density of dislocations is observed. Stress field analysis was performed based on FFT patterns, and the results are shown in Figure 5l. A slight inhomogeneity can be observed in the distribution of tensile stress and compressive stress, which is precisely the origin of the dislocations.

**FIGURE 6:**
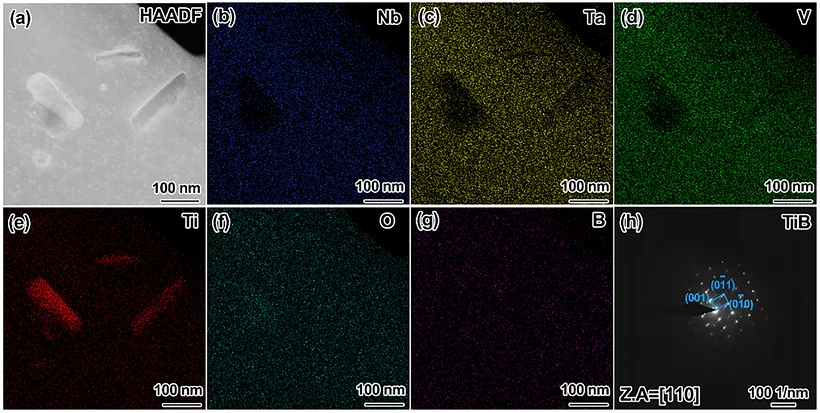
Microstructures of sintered B1 alloy: (a) HAADF TEM image. (b–g) EDS mappings of Nb, Ta, V, Ti, O, and B. (h) SAED pattern obtained from the elongated phase shown in (a).

Figure 6a presents a HAADF image of the precipitated phases in the sintered B1 alloy, in which some whisker‐like precipitates can be observed. Figure 6b–g show the EDS elemental maps for Nb, Ta, V, Ti, O, and B, respectively. Figure 6b–d reveal that Nb, Ta, and V are distributed relatively uniformly. Figure 6e indicates that the whisker‐like precipitates are enriched in Ti. SAED analysis and the indexing procedure performed on one such precipitate confirmed that it corresponds to the TiB phase (Figure 6h).

**FIGURE 7:**
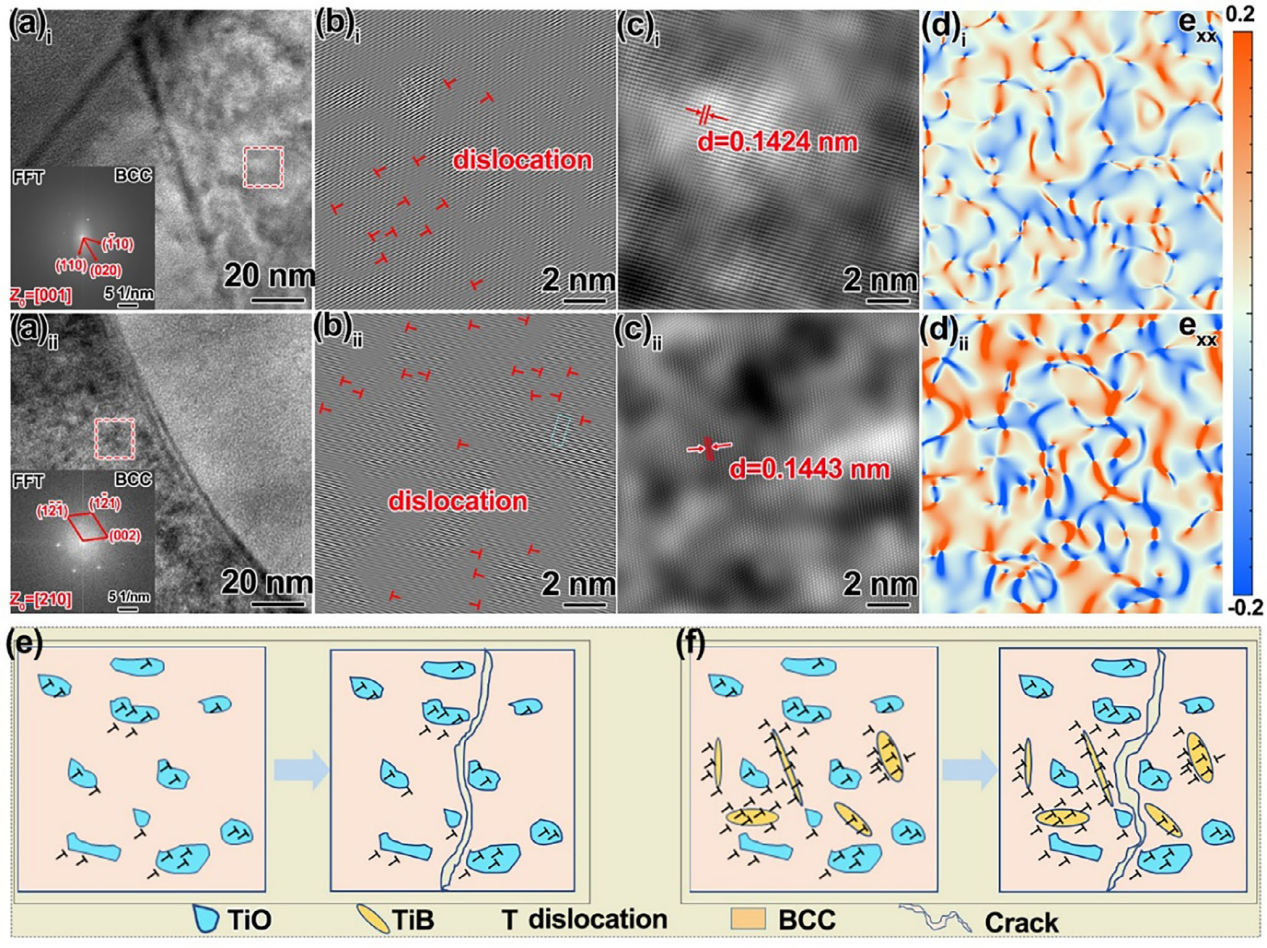
TEM characterizations of sintered B0 (i) and B1 (ii) alloys: (a) High‐resolution TEM (HRTEM) characterization, the illustration shows the fast Fourier transform (FFT) analysis of the selected part. (b) and (c) inverse FFT (IFFT) HRTEM image of selected rectangular part in (a). (d) Comparisons of atomic strain distribution in (b). (e) and (f) respectively represent the fracture diagrams of B0 and B1 alloys.

To further investigate the effect of trace boron on the microstructure of (NbTaTi_1.5_V)_100‐x_B_x_ alloys, TEM micrographs of B0 and B1 alloys were acquired and compared. The results are presented in Figure 7. Indexing of the FFT pattern from the dashed rectangle in Figure 7a_i_ confirmed the BCC structure. The FFT pattern obtained from a high‐resolution image of the B1 alloy is shown in Figure 7aii. The Zone axis of this diffraction pattern is [210]. Indexing of the g‐vectors further confirmed that the region enclosed by the dashed rectangle is the BCC structure. Figure 7bi and Figure 7bii show the IFFT images of B0 and B1 alloys, respectively. A certain number of dislocations can be observed in the IFFT images of both alloys. Notably, the dislocation density in the IFFT image of the B1 alloy is slightly higher than that in the B0 alloy. The interplanar spacings of BCC phase in the B0 and B1 alloys were further measured from IFFT images, and the results are shown in Figure 7ci and cii, respectively. The interplanar spacing of BCC phase in the B0 alloy is 0.1424 nm, while that in the B1 alloy is 0.1443 nm. The stress fields were calculated from the IFFT images for both alloys, and the results are shown in Figure 7di and Figure 7dii, respectively. It is evident that tensile stress dominates in B1 alloy, whereas the stress distribution in B0 alloy is relatively uniform. Figure 7e and f display the fracture morphologies of B0 and B1 alloys, respectively. The fractographs reveal that the presence of TiB in the B1 alloy impedes dislocation motion and leads to dislocation pile‐ups, thereby strengthening the alloy. This strengthening can be attributed to the large lattice distortions, which exert greater frictional resistance to dislocation glide [46].

**FIGURE 8:**
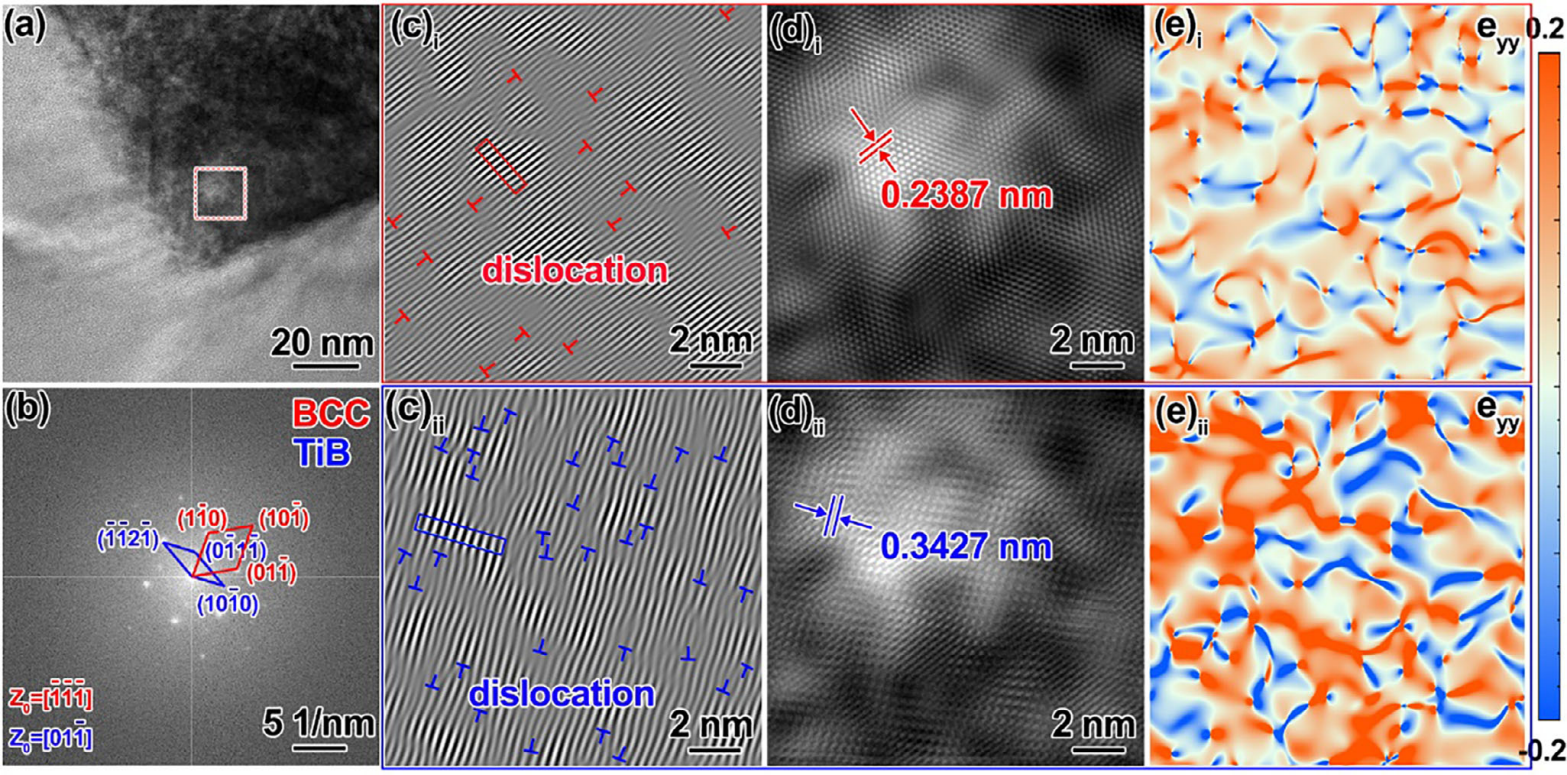
TEM characterizations of B1 alloy. Where: (a) High‐resolution TEM (HRTEM) characterization. (b) the fast fourier transform (FFT) analysis of selected part in (a). (c) and (d) inverse FFT (IFFT) HRTEM image of selected rectangular part in (b). (e) Comparisons of the atomic strain distribution in (d).

To further analyze the microstructure of B1 alloy, the TEM observations results were presented in Figure 8. Figure 8a shows a high‐resolution TEM image of B1 alloy. The FFT patterns were obtained from the selected areas of the HRTEM, and the results are shown in Figure 8b. The indexing procedure applied to the g‐vectors in the FFT patterns reveals that both the BCC and TiB phases coexist in this region. This indicates that boron has reacted with Ti to form TiB, which is consistent with the XRD and EBSD results. Lattice fringe images extracted from the BCC phase and the TiB phase are shown in Figure 8ci and Figure 8cii, respectively. A small number of dislocations are observed within BCC, and they don't exhibit significant accumulation (Figure 8ci). Notably, the dislocation density in the TiB phase is extremely high, as marked in Figure 8c_ii_, which is considered responsible for the significant strengthening of the B1 alloy. This also indicates that plastic deformation can occur in intermetallic compounds [38]. The measured interplanar spacing of BCC is 0.22387 nm (Figure 8di), while that of the TiB phase is 0.34227 nm (Figure 8dii). As shown in Figure 8ei and Figure 8eii_,_ the atomic strain map of vertical normal strain (ε_yy_) in B1 alloy shows a more localized distribution and higher strain values. This result is consistent with the IFFT analysis. High‐density dislocations are precisely the reason for the performance improvement of B1 alloy.

### 3.2 Mechanical Properties of (NbTaTi_1.5_V)_100‐x_B_x_ Alloys

**FIGURE 9:**
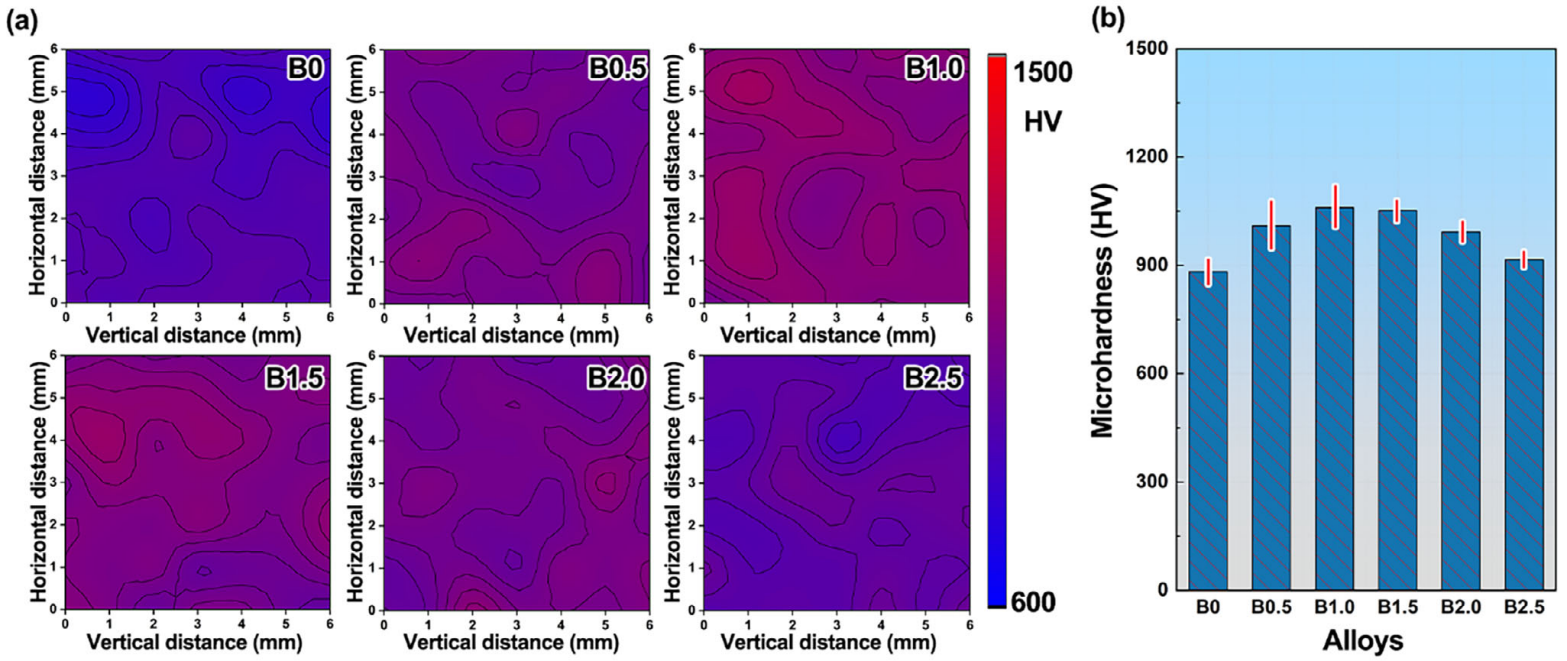
Microhardness distribution contour maps (a) and average value of Vickers hardness (b) for the sintered alloys with different boron content.

Figure 9a shows the microhardness distribution contour maps of sintered (NbTaTi_1.5_V)_100‐x_B_x_ alloys with different boron contents. The B0 alloy shows a relatively uniform microhardness distribution. However, the darker hue in the hardness map indicates lower hardness values. With the addition of boron, the area of bright brown regions in the microhardness map first increases and then decreases. Overall, the total brown area remains larger than of the B0 alloy, suggesting that boron addition enhances the microhardness. The microhardness distribution contour maps provide a direct visual assessment of the hardness uniformity across the alloys. To more clearly reveal the effect of boron content on microhardness, the microhardness values of all alloys were statistically analyzed and are presented as a bar chart in Figure 9b. As can be seen from the figure, the Vickers hardness value of B0.5 alloy increases to 1009 ± 38 HV from the B0 alloy's 881 ± 67 HV with the addition of boron. As the boron content increased, the microhardness first increased and then decreased. At a boron content of 1 at.%, the hardness of B1 alloy reached a peak value of 1060 ± 60 HV, which is 20.4% higher than that of B0 alloy. The hardness of the B1.5 alloy slightly decreases to 1051 ± 29 HV, while that of the B2.5 alloy further drops to 915 ± 21 HV.

**FIGURE 10:**
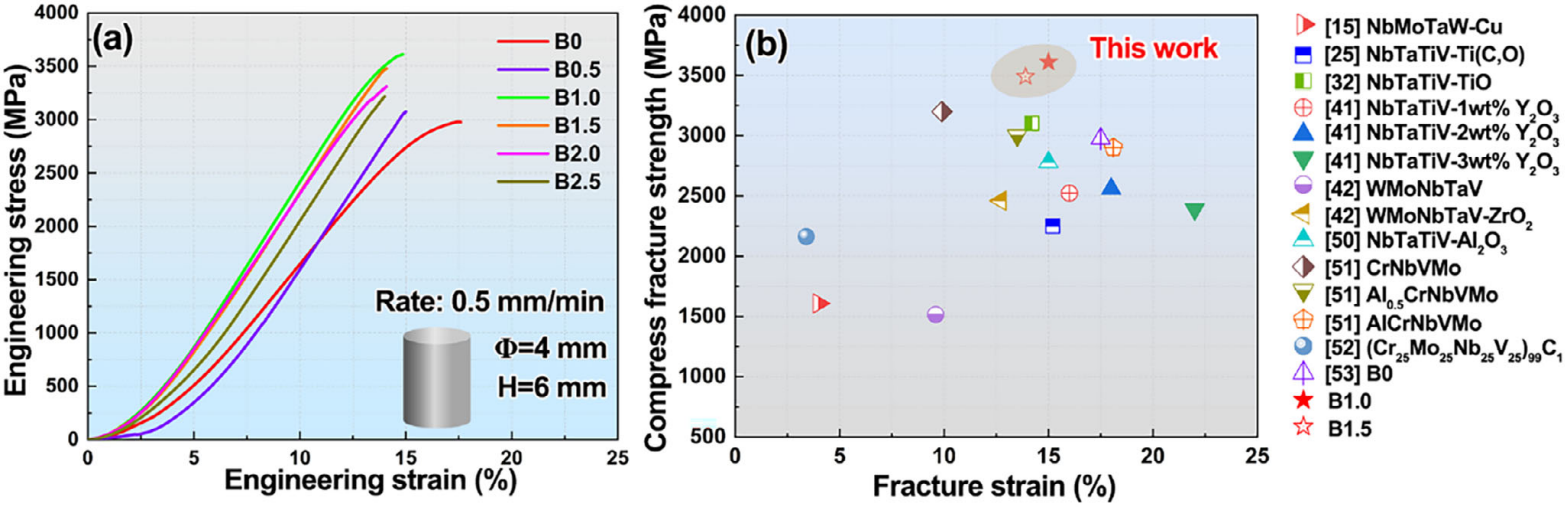
Mechanical properties of (NbTaTi_1.5_V)_100‐x_B_x_ alloys: (a) Compressive stress‐strain curve and ductility of sintered alloys, (b) comprehensive comparison of mechanical properties between (NbTaTi_1.5_V)_100‐x_B_x_ and other representative RHEAs.

Compressive properties are an important criterion for evaluating material properties. Figure 10a illustrates the effect of boron content on the compressive behavior of the (NbTaTi_1.5_V)_100‐x_B_x_ alloys at room temperature. As shown in the figure, a trace amount of boron significantly enhance the fracture strength of (NbTaTi_1.5_V)_100‐x_B_x_ alloys. With increasing boron content, the fracture strength first increases and then decreases. Specifically, the fracture strength of B0 alloy is 2975 MPa, and the fracture strain is 17.6%. After adding 0.5 at.% boron, the compressive fracture strength of B0.5 alloy slightly increased to 3069 MPa, while the fracture strain decreased to 15.0%. When the boron content was increased to 1 at.%, the compressive fracture strength of the B1 alloy increased sharply to 3610 MPa. Compared with B0 alloy, this represents a 21.3% increase in compressive fracture strength, while the fracture strain remains at 14.8% without significant deterioration. As shown in Figure 4, with increasing boron content, the grain refinement effect becomes limited and the grain size tends toward saturation. Accordingly, the B1 alloy exhibits the highest compressive fracture strength. When the boron content was further increased, the compressive fracture strength of the alloys declined. The compressive fracture strength of B2.5 alloy decreased to 3217 MPa, and the compressive fracture strain further decreased to 13.9%. TiB phase acts as a second phase. It pins dislocations and causes stress concentration at the interface. This observation is consistent with the GPA analysis in Figure 2f. This weakens the interfacial bonding, restrains the ability of uniform deformation, and leads to a continuous decrease in fracture strain. In addition, changes in the degree of lattice distortion also cause significant fluctuations in the elastic modulus [40, 47, 48, 49]. Furthermore, this study compares the compressive properties of (NbTaTi_1.5_V)_100‐x_B_x_ alloys with those of other RHEAs reported in the literature [15, 25, 32, 41, 42, 50, 51, 52, 53]. The results are shown in Figure 10b. The boron‐containing alloys prepared in this work exhibit both relatively high room‐temperature compressive fracture strength and relatively high fracture strain. The above results indicate that the addition of an appropriate amount of boron is an effective strategy for strengthening the (NbTaTi_1.5_V)_100‐x_B_x_ alloys.

**FIGURE 11:**
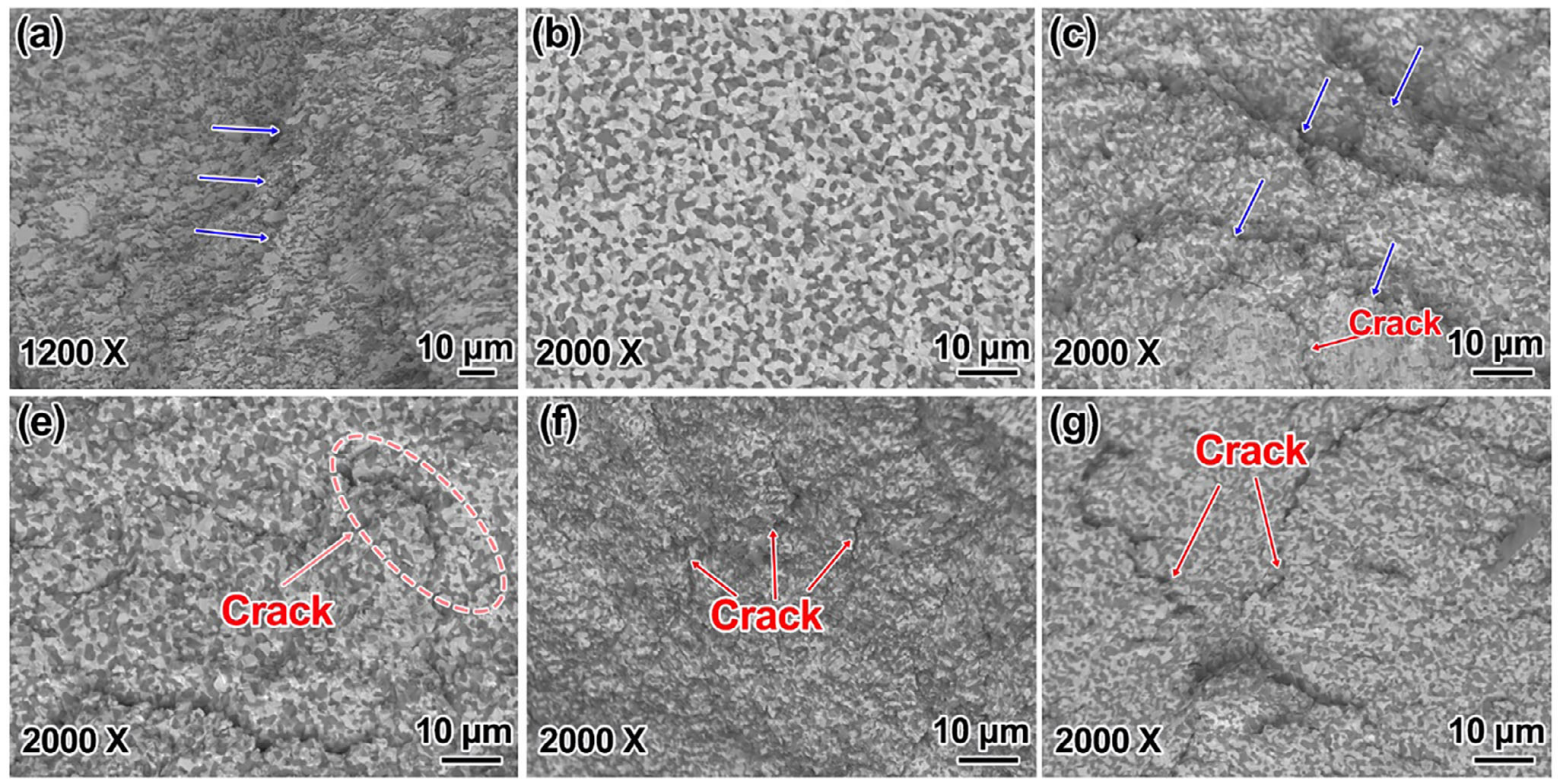
Compressive fracture morphologies of (NbTaTi_1.5_V)_100‐x_B_x_ alloys at room temperature.

Figure 11 shows the compressive fracture morphologies of sintered (NbTaTi_1.5_V)_100‐x_B_x_ alloys. Overall, the fracture morphologies of the alloys with different boron contents all exhibit brittle fracture characteristics under room‐temperature compression, which is consistent with the fracture behavior of BCC‐structured alloys reported in the literature [51, 54]. Figure 11a shows that the room‐temperature compression fracture surface of B0 alloy presents an uneven morphology. The blue arrows indicate pronounced height steps on the B0 fracture surface, providing evidence that the crack front was repeatedly deflected. This tortuous crack path dissipates more energy, which corresponds to the largest compressive fracture strain. The compressive fracture morphology of B0.5 alloy is relatively smooth, as shown in Figure 11b. Figure 11c reveals the fracture surface of B1 alloy also exhibits an uneven morphology, as marked by the blue arrow. In addition, some cracks are also observed on the compressive fracture morphology of B1 alloy. With increasing boron content, an increasing number of cracks are detected on the fracture morphology of B1.5, B2 and B2.5 alloys. It is shown in Figure 11e,f, and g, respectively. This indicates that the compressive fracture strain of the alloy decreases with increasing boron content, which is consistent with the room‐temperature compressive stress‐strain curves.

### 3.3 Strengthening Mechanisms

In this study, (NbTaTi_1.5_V)_100‐x_B_x_ alloys were synthesized by ball milling followed by SPS. TiO and TiB phases formed in situ. The latter generates a high density of dislocations, providing substantial additional strengthening to the alloy. Therefore, the strengthening of the present alloys arises from the superposition of multiple contributions, including the intrinsic strength of the matrix (σ_0_), solid solution strengthening (σ_*ss*_), precipitation hardening due to TiO (σ_*TiO*_), dislocation strengthening (σ_ρ_), grain boundary strengthening (Δσ_*gb*_) as described by the Hall–Petch relationship, and load transfer strengthening (σ_*Load*_) from the matrix to the reinforcements. The yield strength (σ_*y*_) of B1 alloy can be estimated by summing the contributions from each mechanism, as expressed by the following equation:

(1) $$\sigma_{y}=\sigma_{0}+\sigma_{\rho }+\sigma_{ss}+\Delta \sigma_{gb}+\sigma_{Orowan_{TiO}}+\sigma_{Load}$$

where σ_0_ represents lattice friction stress. The intrinsic strength (σ_0_) was calculated from the annealed yield strengths of the pure metals Nb, Ta, Ti and V using the following formula [13, 55]:

(2) $$\sigma_{0}=\sum c_{i}\sigma_{iy}$$

where *c_i_* is the atomic percentage of each element in the alloy, and σ*_iy_* is the yield strength of the annealed pure metal. In the present work, the yield strengths of annealed pure Nb, Ta, Ti and V are 240 MPa, 345 MPa, 195 MPa, and 310 MPa, respectively [56]. Therefore, the intrinsic strength of the PM B1 RHEA was calculated to be 198 MPa based on Equation (2).

A large number of dislocations are observed in Figure 8, so the reinforcement caused by dislocations cannot be ignored. Dislocation strengthening (σ_ρ_) can be described by the Taylor‐hardening relation [57, 58], and the value can be calculated by Equation (3):

(3) $$\sigma_{\rho }=\alpha MGb\sqrt{\rho }$$

where α is an empirical constant (0.38 for a BCC matrix [32]), *M* is the Taylor constant (with a value of 2.9) [59], and ρ refers to total dislocation density [60]. The magnitude of the Burgers vector b is equal to *b* = (3/2)^(1/2)^a [13, 55], and a is the lattice constant of the matrix [29]. The values of *G* (52.03 GPa) and *v* (0.368) are approximately used according to the literatures [25, 29, 61]. According to the transmission analysis, a is 0.2387 nm. The parameters in the calculation are listed in Table 2. The XRD patterns can be attributed to an increase in dislocation density [32, 62]. The dislocation density can be calculated by determining the crystallite size (D) using the Scherrer equation [63]:

(4) $$D=\frac{k\lambda }{\beta \cos\theta }$$

(5) $$\rho =\frac{1}{D^{2}}$$

where *k*(0.9) is Scherrer's constant, λ (0.154 nm) is the X‐ray wavelength, *β* is the full width at half maximum, and θ is the Bragg's diffraction angle. The value of ρ is 5.67 × 10^15^ m^−2^, and the calculated strength contribution of dislocations (σ_ρ_) is 892 MPa.

**TABLE 2:** Values of the parameters used [25, 29, 32, 59, 64].

Parameter	*G* (GPa)	*b* (nm)	*ν*	*M*	α	*a* (nm)	*β*(°)	2θ
Value	52.03	0.2067	0.368	2.9	0.38	0.2387	0.63531	39.522

Elements form a BCC solid solution structure. Therefore, solid solution strengthening should be taken into consideration. This calculation is based on the principle of elastic interaction between local lattice distortions and the stress fields of dislocations [65], and the yield strength caused by the solid solution strengthening (Δσ_ss_) can be expressed as [66, 67]:

(6) $$\Delta \sigma_{\mathrm{ss}}={\left(\sum \Delta {\sigma_{i}}^{3/2}\right)}^{2/3}$$

(7) $$\Delta \sigma_{i}=AGf_{i}^{4/3}{C_{i}}^{2/3}$$

(8) $$f_{i}=\sqrt{\delta_{G_{i}}^{2}+\beta^{2}\delta_{r_{i}}^{2}}$$

where: A is a fitting constant of the value of 0.04, and *f_i_* is the misfit parameter for the HEA [56]. $\delta_{G_{i}}$ is the shear modulus mismatch, $\delta_{r_{i}}$ is the atomic size mismatch between the solute and the solvent. The coefficient *β* is about 9, which is associated with activated dislocation type [56].

The structural feature of BCC solid solution is that each solute atom is surrounded by 8 atoms to form an atomic cluster of 9 atoms. The modulus and atomic size differences between solute and solvent cause local lattice distortion near the solute. Equations ((9), (10), (11), (12)) help estimate this distortion for each element.

(9) $$\delta_{r_{i}}=\frac{9}{8}\sum C_{j}\delta_{r_{ij}}$$

(10) $$\delta_{G_{i}}=\frac{9}{8}\sum C_{j}\delta_{G_{ij}}$$

(11) $$\delta_{r_{ij}}=2\left(r_{i}-r_{j}\right)/\left(r_{i}+r_{j}\right)$$

(12) $$G_{ij}=2\left(G_{i}-G_{j}\right)/\left(G_{i}+G_{j}\right)$$

where *C_j_* is the atomic fraction of jth element in the alloy, $\delta_{r_{ij}}$ is the atomic size difference of ith element and *G_ij_* is the atomic modulus difference of ith element and jth element. The atomic radius, shear modulus, and yield stress of pure metals are listed in Table 3.

**TABLE 3:** Shear modulus (*G* in GPa), atomic radius (r in pm), and yield strength (σ_0.2_ in MPa) of pure metals [67].

Element	Nb	Ta	Ti	V
*G* (GPa)	37.5	69.2	45.6	46.6
r (pm)	1443	143	145	132
σ_0.2_ (MPa)	240	170	195	310

**FIGURE 12:**
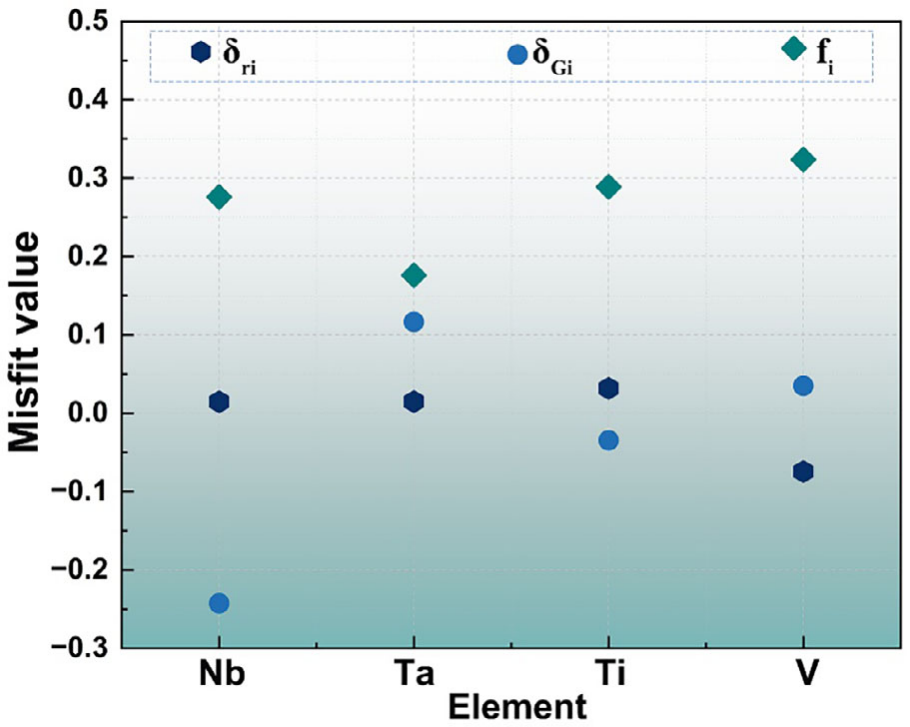
Calculated effect of solid solution strengthening of (NbTaTi_1.5_V)_100‐x_B_x_ alloys.

To quantitatively analyze the contribution of each element to the strengthening of the alloy and provide a qualitative explanation, the values of calculated mismatch parameter f_i_, lattice mismatch parameter δ_Gi_, and atomic size mismatch parameter δ_ri_ are plotted in Figure 12. It is clearly shown in the figure that the absolute values of f_Ti_ and f_V_ are greater than f_Ta_ and f_Nb_, which indicates that Ti and V play a major role in the solid solution strengthening process. It is calculated that the solid solution strengthening in B1 alloy is about 367 MPa.

A large amount of TiO is precipitated in B1 alloys, which could impede dislocation motion and thereby strengthen the alloys. The contribution of precipitation strengthening of TiO could be estimated using the Orowan dislocation bypass mechanism. The Orowan strengthening mechanism can be mathematically described by the following equation [68, 69].

(13) $$\sigma_{\mathrm{or}}=M\frac{0.4Gb}{\pi \lambda }\cdot \frac{\ln(2\bar{r}/b)}{{\left(1-v\right)}^{1/2}}$$

where: *M* (with a value of 2.9) represents the average orientation factor for the BCC polycrystalline matrix [59]. *G* denotes the shear modulus of the material. *v* is the Poisson's ratio. *b* is the Burgers vector of the matrix. The parameter $\bar{r}$ is defined as (2/3)^1/2^ *r*. Here, *r* is the mean radius of the precipitate. *λ* is the interspacing of a precipitate, and it can be calculated as follows [70]:

(14) $$\lambda =2\bar{r}\left(\sqrt{\frac{\pi }{4f}}-1\right)$$

where: *f* is the volume fraction of the precipitate. The parameters are listed in Table 4. According to Equations (13) and (14), the Orowan reinforcement in the alloy is about 10 MPa.

**TABLE 4:** Values of the parameters used [25, 29, 59, 64].

*M*	*G* (GPa)	*b* (nm)	*ν*	*r* (µm)	*λ*	*f*
2.9	52.03	0.2067	0.368	0.93	1.121	0.26

Given the limited influence on the dislocation movement, it is crucial to consider the contribution of reinforcements on σ_*Load*_ [71]. This contribution can be calculated as follows:

(15) $$\sigma_{Load}=0.5\sigma_{m}lV_{TiO}\%$$

where σ_*m*_ is the yield strength of matrix material. Here, it is taken as 1064 MPa [61]. *l* is the aspect ratio of TiO. It is worth noting that TiO is mostly equiaxed, so the value of *l* is approximately 1, while *V* represents the volume fraction of TiO. After calculation, the load transfer enhancement is 138 MPa.

**FIGURE 13:**
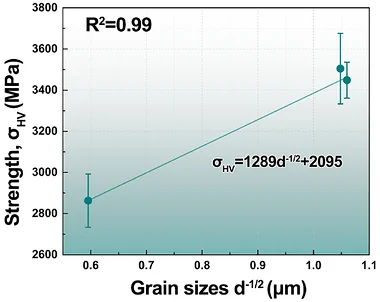
Hall–Petch relationship curve between the stress and grain sizes of (NbTaTi_1.5_V)_100‐x_B_x_ alloys.

In this work, (NbTaTi_1.5_V)_100‐x_B_x_ alloys with a fine‐grained microstructure were prepared by MA and SPS. Moreover, the addition of boron significantly refines the grains. The yield strength increase caused by grain size difference can be expressed as Equation (16) [13, 32, 55, 72, 73].

(16) $$\Delta \sigma_{\mathrm{gb}}=k_{\mathrm{HP}}d^{\left(-1/2\right)}$$

where *k*_HP_ is Hall–Petch coefficient, *d* is the grain size of (NbTaTi_1.5_V)_100‐x_B_x_ alloys. Zhan et al. [13] and Jia et al. [74] have demonstrated that the empirical relationship σ_HV_ = 3.295H is also applicable to HEAs and MEAs, and it was applied to the HfMoNbTaTi RHEA and Ti_43.3_V_28_Zr_14_Nb_14_Mo_0.7_ MEA, respectively. Where σ_HV_ is the strength converted from hardness in MPa, *H* is the hardness value in HV. Other studies have also reported the determination of *k*_HP_ by fitting the strength data of various alloys [32, 75]. In the present work, to accurately determine the *k*_HP_ values, the complete Hall–Petch formula and measure hardness. The specific calculation procedure is outlined in Figure 13. This method has been reported by Zhan et al. [13] and successfully applied to the HfMoNbTaTi RHEA. According to Figure 13, *k*_HP_ = 1289 MPa µm^1/2^. The increment of yield strength caused by grain refinement is calculated to be 1496 MPa.

**FIGURE 14:**
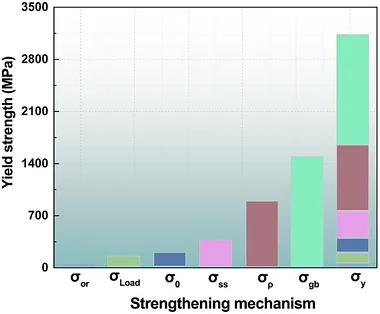
Contributions of different strengthening mechanisms in the B1 alloy during compression at room temperature.

The contributions from the various strengthening mechanisms were quantified, and the results are shown in Figure 14. Grain boundary refinement strengthening (Δσ_*gb*_) is the dominant mechanism, accounting for 48.2% of the total strengthening increment. This enhancement stems from boron‐induced grain refinement, as corroborated by grain size statistics shown in Figure 4. In second place are the dislocation strengthening (σ_ρ_) and solid solution reinforcement (σ_*ss*_) with a share of 28.7% and 11.8%, respectively. Additionally, the intrinsic strength (σ_ρ_) contribution is about 6.4%.

**FIGURE 15:**
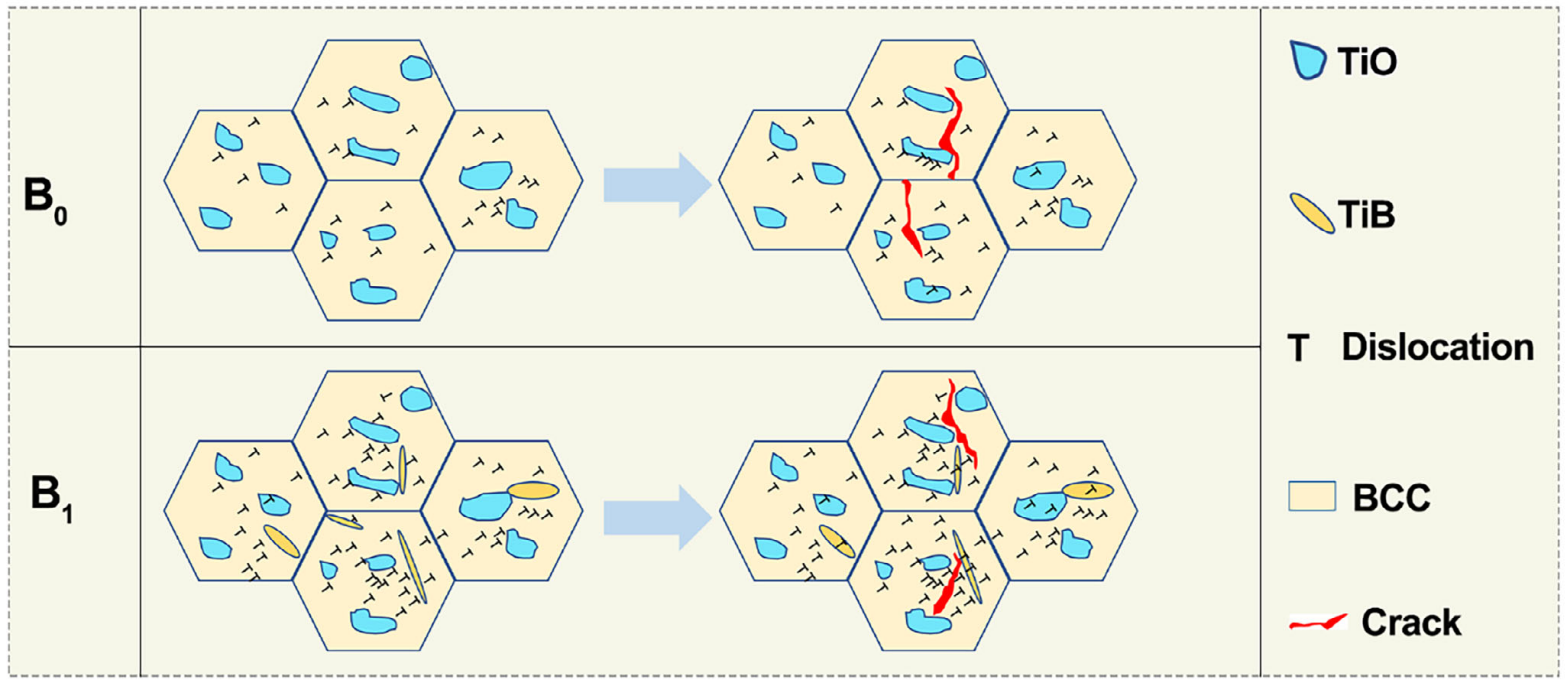
Schematic diagram of fracture mechanism of B0 and B1 alloys.

Taking above together, the microstructure and fracture process of B0 and B1 alloys are shown in Figure 15. In B1 alloy, the TiB reinforcing phase is strengthened through dislocation strengthening and crack deflection effects. Meanwhile, the grain boundary strengthening caused by the addition of boron effectively enhances the compressive yield strength of the alloy [58].

## 4 Conclusions

In this study, (NbTaTi_1.5_V)_100‐x_B_x_ alloys with different boron contents were prepared by SPS. The effects of different boron content on the microstructure and mechanical properties of (NbTaTi_1.5_V)_100‐x_B_x_ alloys were investigated. The performance of B1 alloy was strengthened by the dual strengthening effect of *in‐situ* generated TiO and TiB through the addition of boron. Additionally, the strengthening mechanisms were analyzed using strengthening models, leading to the following conclusions:

1. The (NbTaTi_1.5_V)_100‐x_B_x_ alloys prepared by SPS were composed of a BCC matrix and *in‐situ* TiO and TiB reinforcement phases. The integrated ball milling and sintering process inhibited the agglomeration and growth of the reinforcement phase, achieving a uniform distribution of reinforcing phase. Meanwhile, the introduction of trace amounts of boron significantly refined the grains. The average grain size of B1 alloy was only 0.91 µm, representing a 67% reduction compared with that of the B0 alloy (2.82 µm). This provided a favorable microstructural foundation for the synchronous strengthening of the alloy.
2. With the increase of boron content, the compressive strength of the alloys first increased and then decreased. With the addition of 1 at.% boron, the compressive fracture strength of B1 increased sharply to 3610 MPa, representing a 21.3% increase compared with that of the B0 alloy, while a compressive fracture strain of 14.8% was maintained. The microhardness rose from 881 ± 67 HV to 1060 ± 60 HV, with an increase of 20.4%. The outstanding mechanical properties stemmed from the synergistic strengthening of TiO and TiB, with the TiB phase in particular impeding dislocation slip and promoting dislocation pile‐ups.
3. This study employed a variety of strengthening methods to enhance B1 alloy. The addition of boron refined the grains, thereby achieving grain‐boundary strengthening. Among these mechanisms, the refinement of grain boundaries was the main mechanism, accounting for 48.2% of the total strengthening increment. In addition, TiB and TiO particles induced dislocation proliferation and hindered crack propagation, thereby significantly strengthening B1 alloy. The contribution of this dislocation reinforcement mechanism was approximately 28.7%.

## Author Contributions

**Da Wu**: Formal analysis, writing – original draft, investigation. **Bo Li**: Conceptualization, methodology, writing – review and editing, supervision, funding acquisition. **Yuzhi Shi**: Methodology. **Cong Li**: Investigation, writing – review and editing. **Eryong Liu**: Methodology. **Xiaohu Hou**: Methodology. **Yimin Gao**: Supervision. **Tao Wu**: Methodology. **Pucun Bai**: Writing – review and editing. **Chenyu Liang**: Methodology.

## Conflicts of Interest

The authors declare no conflicts of interest.

## Data Availability

The data that support the findings of this study are available from the corresponding author upon reasonable request.
